# A Postphenomenological Perspective On the Changing Nature of Work

**DOI:** 10.1007/s10606-022-09447-2

**Published:** 2022-09-06

**Authors:** Anastasia V. Sergeeva

**Affiliations:** School of Business and Economics, Vrije Universiteit Amsterdam, De Boelelaan 1105, 1081 HV Amsterdam, the Netherlands

**Keywords:** Postphenomenology, Changing nature of work, Distributed work, Technology, Materiality, Telemedicine, Digital transformation

## Abstract

In this essay, I take a postphenomenological perspective on tracing work transformation during the pandemic, arguing that this perspective helps develop novel sensitivities to the nature of work. Postphenomenology brings into high relief the view on work as reliant on sensory performances and embodied relations, complementing already rich accounts of work being reliant on discursive interactions, social order, and spatiality. The focus of postphenomenology on ‘non-neutrality’ and the multistability of technology provides a useful lens for revealing a multiplicity of changes, encompassing both augmentations and reductions of work experiences and evaluating their consequences for the actors involved. Finally, its attention to the transparency of technology amidst the embodied experiences gives a handle on the role of materiality in the performance of work and may be taken up as informing design efforts. A case study vignette of physiotherapy work during lockdown is offered as an illustration of applying some of the postphenomenological ideas.

## Introduction

You’ve actually lost a sense.

If you're going blind, you've got to be better at hearing*.*

—Interview with a physiotherapist

The introduction of the COVID-19 related measures across countries resulted in multiple restrictions of how regular work activities could be performed. Major conversations followed both in popular media and research about the nature of work, the effect of new conditions on productivity and work conditions, as well as the future of work beyond the pandemic. Commentaries and emerging studies of work during the lockdown provided a variety of assessments of the crisis’s impact: while initial sentiments were focusing on highlighting the disruptions of e.g., work-life balance, productivity, or mental health (Feng and Savani, 2020; Haag, 2020; Toniolo-Barrios and Pitt, 2021), later more nuanced voices also brought up potential benefits, such as speeding up digital transformation, enjoying more time with family, and a decrease in the number of ‘useless’ meetings (Davison, 2020; Gkeredakis et al., 2021; Papagiannidis et al., 2020). Later conversations shifted to discussing strategies to cope with the changing world (Greenberg and Hibbert, 2020; Hunt, 2020; Orlikowski and Scott, 2021). This paper suggests engaging with these questions using a postphenomenological perspective, offering it as a valuable lens for understanding the changing nature of work and acknowledging its bodily, fluid, and technologically mediated nature.

I suggest that a postphenomenological reading of work transformation is helpful in several ways. First, it brings into high relief the view of work as being reliant on sensory performances and embodied relations, complementing already rich accounts of work being reliant on discursive interactions, social order, and spatiality (e.g., Bardram and Bossen, 2005; Hindmarsh and Heath, 2000a; Luff et al., 2000; Suchman, 1987). Second, its focus on the perceptual transformation and its duality, i.e., encompassing both augmentations and reductions, provides a useful lens for tracing fluidity of work in times of crisis and evaluating their emotional significance for actors involved. Finally, its attention to transparency and multistability of technology gives a handle on the role of materiality in the performance of work and may be taken up as informing design efforts.

In what follows, I outline this perspective by first introducing several tenets of postphenomenological theory and situating them in relation to existing approaches in computer supported cooperative work (CSCW) and human-computer interaction (HCI) communities. Specifically, I offer several concepts, such as embodied relations, augmentation, reduction, multistability, and transparency as useful ones for understanding the shifts in work during the pandemic. A case study vignette of physiotherapy work during lockdown is then offered as an illustration of applying some of the postphenomenological ideas to the analysis of work. I conclude with the implications of this perspective for CSCW research and understanding of the changing nature of work during the pandemic and beyond.

## Postphenomenology as a Lens on the Changing Nature of Work

Perhaps the most helpful way to introduce postphenomenological theory is to explain its origin and key points of difference when compared to classical phenomenology. In fact, postphenomenology has been developed as a criticism of phenomenological thinking, emerging from the growing dissatisfaction with the latter’s overly transcendental treatment of technology and romanticism in relation to the authentic human lifeworld. Since the differences between phenomenology and postphenomenology are rarely explicitly discussed in CSCW or related communities, a short detour to this branch of philosophy is warranted.

Phenomenological tradition is often seen as an underpinning of the broader branch of philosophy, ‘philosophy of technology’, that occupies itself with the role of technology in human and societal affairs. Early phenomenologists Edmund Husserl, Martin Heidegger, and Maurice Merleau-Ponty, while not necessarily focusing on theorising technology as their central theme, have offered the first crucial components for thinking about human-technology relations. Their focus on the ontological primacy of ‘being-in-the-world’ and emphasis on the practical nature of engaging in mundane activities have also offered a novel way to think about objects and tools as something inseparable from situated activity. Because their ontology was different from Cartesian dualism and provided an explicit way to think about the role of objects relationally and as part of practice, the work of these philosophers has influenced multiple fields related to computing and systems design, such as CSCW, HCI, and information systems (IS) (e.g., see Dourish (2001) for an overview).

Specifically, the work of Martin Heidegger has made a lasting impact on studies of computing. Heidegger’s concepts such as ‘ready-to-hand’, ‘thrownness’, ‘equipment’, as well as his analysis of how technology only appears to us in moments of breakdown, illustrated by the hammering example, were generative for developing a counter perspective to a dominant rationalistic paradigm for computing (Winograd and Flores, 1986), and foundations for the embodied interaction in HCI (Dourish, 2001). In CSCW, the focus of ethnomethodological studies on concrete practices and emphasis on practical action and intelligible ordinary conduct reflect a similar ontological commitment (Crabtree et al., 2012; Fischer et al., 2013; Heath and Luff, 1992). And in IS, Heidegger’s work has been utilized to re-think dominant cognitive approaches to ‘technology adoption and appropriation’ (Orlikowski, 1992; Riemer and Johnston, 2014). However, it is Heidegger’s later, post-war phenomenology (sometimes dubbed the *Kehre* period) where he is much more explicitly addressing the question of technologies, and specifically, those that are based on scientific inventions that have become a point of critique in the philosophy of technology, but that is less often enrolled in discussions in other disciplines.

In two influential essays ‘The Question Concerning Technology’ (Heidegger, 1977) and ‘The Memorial Address’ (Heidegger, 1966), Heidegger is often interpreted to be expressing his view on technology as a *profoundly alienating force* that is bringing humans away from their authentic being and lifeworld (Verbeek, 2005). Referring to such industrial technologies as hydroelectric plants and atomic bombs, Heidegger is deeply concerned with the implications that such technologies create for humanity, turning nature into ‘standing reserve’, transforming nature and people into resources to be exploited. It is crucial that the technology that he refers to in those essays are ‘modern’ technological means, developed as a result of advance of ‘modern physics’,^1^ and which he sees as different from such more ‘older handwork’ technologies as a hammer; an example of technology that is typically enrolled in HCI or IS studies (e.g., Dourish, 2001; Riemer and Johnston, 2017; Riemer and Johnston, 2014; Winograd and Flores, 1986).

Postphenomenology, in turn, emerges as a response to Heidegger’s ‘alienation’ thesis and ‘nostalgic’ Romanticism, suggesting that an alternative and less monolithic take on modern technologies would be more generative. Postphenomenology makes it as its program to not presuppose technology’s universal power to somehow rob humanity of its natural authentic lifeworld, but instead to focus on examining ‘technology-in-the-particular’, the situated uses of specific technologies in specific practices. The program of postphenomenology is to turn to empirical studies of technologies not from the perspective of ‘alienation’, but from the perspective of ‘mediation’: how technologies are and have always been constitutive of being human in the first place.^2^ Postphenomenology does continue to emphasise the inseparability of perception and action, as well as the ontological primacy of experience and ‘being-in-the-world’, but in contrast to classical phenomenology does so with the explicit recognition that almost no activities in the world are free from technologies. In fact, Ihde’s (1990) introduction to postphenomenology starts with the imaginative detour into the primary experience of humanity in the ‘Garden’ to make a point that ‘virtually every area of praxis implicates a technology’ (p. 20).

One of the focal points of postphenomenology that may be also informative for CSCW (and of work changes during the lockdown) is the explicit analysis of various structural features of human-technology relations: the analysis of how situated experiences differ under the conditions of the presence or absence of technological mediation. This analysis zooms into the specifics of the transformations of perception, trying to distinguish the subtle but consequential changes in sensory performance that are brought forward by mediation. Ihde (1979) illustrates such an analysis using an example of feeling the tooth with a finger in comparison with using a dentist probe:

I can, of course, feel the tooth with my finger. In this case I do get the sense of the tooth’s hardness, its texture and more besides. But compared to the sense of the tooth through the probe, I now note that something is missing as well. The probe not only extended my embodiment, it amplified certain characteristics of the tooth. Through the probe I actually get a better sense of the hardness and softness of the tooth surfaces, a finer discrimination. The probe gives me what, compared to the fleshy finger, are micro-features of the tooth’s surface. Thus, part of the amplification of the instrument also reveals micro-features only partly available, or perhaps not at all available to my finger. (p. 20–21)

The point about the dual role of technology—both augmenting some sensory experiences and reducing others—is referred to as the *amplification-reduction* principle and is illustrative of a non-straightforward, or what is also referred to as ‘non-neutral’, transformation of human perception and action that technology makes possible.

Postphenomenological vocabulary may become useful for research on work in the way it attends to the materiality of technological features in shaping, mediating, and participating in the flow of experience. The materiality of technology is suggested to not be assessed on its own terms but rather through such concepts as *multistability* and *transparency.* The notion of multistability aims to embrace both the constraining character of technology and its open-endedness in terms of allowing multiple uses, emerging from specific combinations of users’ embodied skills, techniques of use, and cultural contexts of practice. Multistability implies that the ‘same’ technology may exhibit a variety of quite different ‘stabilities’: variations of their materialities and uses that come to surface when we inquire into practices of their use in particular historical settings. To uncover those stabilities, an examination must be made of the ‘active perceptual engagements’ with those technologies, that is how they are enrolled by the bodily active humans. Ihde (2009) illustrates this point through a historical analysis of archery practice: while archery in various cultures can be said to rely on the ‘same’ technology as a bow, bowstring, and an arrow; the actual materialities of bows and arrows (e.g., curvy, long, heavy or light, different composite materials) differed across English, Mongolian, and Chinese cultures, depending on the practice of warfare and style of firing e.g., while at a gallop or standing still (Ihde, 2009, pp. 17–19).

The concept of *transparency* is another way of including the materiality of technology into the analysis of embodied relations—when the qualities of technology are such that they help attain transparency, technology fades to the background of users’ awareness and instead becomes a natural, almost cyborg-like, extension of bodily senses. The point about transparency bears heritage to the ‘withdrawal’ point of Heidegger, i.e., tools fading from focal awareness. However, transparency in postphenomenology is also used to highlight another dimension: technological mediation of embodied relations with the world, Ihde argues, also carries with it a distinct emotional component, in a sense of both striving for the new extended bodily capacities made possible by tools, but simultaneously rejecting the limits that put a reduction on ‘naked’ bodily capacities. Ihde writes:

The desire is, at best, contradictory. I want the transformation that technology allows, but I want it in such a way that I am basically unaware of its presence. I want it in such a way that it becomes me. Such a desire both secretly *rejects* what technologies are and *overlooks* the transformational effects which are necessarily tied to human-technology relations. This illusory desire belongs equally to pro- and anti-technology interpretations of technology.

The desire is the source of both utopian and dystopian dreams. The actual, or material, technology always carries with it only a partial or quasi-transparency, which is the price for the extension of magnification that technologies give. In extending bodily capacities, the technology also transforms them. In that sense, all technologies in use are non-neutral. They change the basic situation, however subtly, however minimally; but this is the other side of the desire. The desire is simultaneously a desire for a change in situation—to inhabit the earth, or even to go beyond the earth—while sometimes inconsistently and secretly wishing that this movement could be without the mediation of the technology. (Ihde, 1990, p. 75)

The commitment to include the materiality of technology into consideration, and at the same time assessing it from the vantage point of perceptual experience is what can make a postphenomenological approach useful for considering technology’s role in work, offering a way of accounting for the interplay of material and human agency. For example, postphenomenology could be useful for understanding the emotional stances and ambivalence that users may experience towards the tools that are offered for mediating their activities with distinct bodily nature, e.g., drones for manipulating objects in space (Rauch and Ansari, 2022), robots for performing surgery (Sergeeva et al., 2020) or augmented reality in industrial work (De Carvalho et al., 2018). In addition, some of postphenomenology’s main ambitions are to also bring the role of philosophy into guiding the responsible design of artefacts. These purposes and empirical character bring postphenomenology close to the main purposes of the CSCW field. Its interest in interrogating the human-world relations may offer a complementary insight that builds on Heideggerian ontology but extends and refines it with a more explicit analysis of the changes brought forward by mediation.

Postphenomenology shares with much of the CSCW research its commitment to understand and portray the situated conduct in all its richness (Hindmarsh and Heath, 2000b; Luff et al., 2000; Star and Strauss, 1999; Suchman, 1993). Where I suggest it can extend the CSCW studies is in joining it to further examine bodily and perceptual performance and especially its transformation as the unit of analysis. The bodily performance of work has been brought into high relief during the pandemic, exposing its perhaps previously underappreciated nature—due to the social distancing measures, many of the traditional bodily performances on which work had been reliant—became impossible. The shift in work activities during the pandemic can thus become a revelatory occasion to examine the transformation of the embodied relations in the performance of work and reconfigurations of technological mediation. It also can teach us about how subsequent experimentation with technologies and bodily skills exposed multistability of everyday technologies that became repurposed to ensure work continuity.

In what follows, I offer a physiotherapy vignette as an illustration of what kind of coping strategies were taken up by actors to deal with the breakdowns of embodied relations. I draw on postphenomenological ideas to first illustrate how the forced move to provide remote care created reductions of embodied relations and few augmentations it offered. I then report on the coping strategies of physiotherapists, showing how they repurposed general communication technology, recalibrated senses, and enrolled mundane artefacts and routines to relate to their patients online. I then explain how those strategies allowed to temporarily sustain operations, provided an opportunity to experience the performance of telecare first-hand and as a result triggered the emergence of alternative stabilities of everyday tools for the purpose of work. Eventually, physiotherapists arrived at reflections that telecare could potentially become a viable extension rather than a replacement for their traditional practice.

## Case Study Vignette: Physiotherapy during Lockdown

### Setting and Informants

The vignette features insights from in-depth semi-structured interviews with 10 physiotherapists working in the Netherlands, focusing on their experiences with having to provide remote care to their patients.^3^ Because of the situation around the COVID-19 crisis, only four of the interviews took place face-to-face, and the remaining six were held via video conferencing. Informants were identified via professional social media, such as LinkedIn, as well as personal social networks, and then via the snowballing technique for inviting new potential interviewees. In selecting informants, an important criterion was to find those physiotherapists who engaged in remote care in response to the lockdown and had a substantial enough direct experience of treating patients at a distance. During interviews, we focused on eliciting descriptions of what the nature of changes were for physiotherapists in how they could perceive patients, perform care, and their broader reflections on how they perceived their work under new conditions. Interviews lasted 40 minutes on average and were fully transcribed afterwards. We used thematic coding techniques to analyse the data.

### Changes in Embodied Relations with Patients

The major reduction for physiotherapists was the loss of haptic perception and ability to directly manipulate and direct patient movements. Almost all physiotherapists shared the sentiment of being handicapped by not being able to engage with their patients in a haptic way. As one physiotherapist noted:

[The biggest difficulty is]: the inability to touch a patient. ‘Cause physiotherapists touch their patients. We touch our patients all the time. And that is the biggest change. Trying to become creative in how to treat them without touching them.’ (Physiotherapist 4)

Another one similarly shared: ‘You’ve actually lost a sense. So, you can no longer feel and do things [with your hands].’ (Physiotherapist 10). Another physiotherapist lamented: ‘My profession, and what I love about it, is of course hands-on, and that is not possible to perform through a power cord.’ (Physiotherapist 6)

While the loss of haptic perception was most frequently deplored, no longer being able to meet their patients face-to-face made many physiotherapists aware of how much they relied on the whole sensory apparatus in their work and how important it used to be to perceive the patients with the ‘whole’ of embodiment. One physiotherapist reflected on the value of this holistic sensing:

The most ideal situation is to just talk with someone face-to-face, because you will get the whole picture: how is someone sitting? Does he tap his feet? Does he snap his fingers? So, you lose a lot when it comes to what you could see and feel with a patient. Thus, in this case, you have to focus extra on the words that someone is using. (Physiotherapist 3)

In terms of augmenting the embodied experience of care, the physiotherapists did not encounter much of an upgrade. Among minimal augmentations were the possibilities of seeing the patient in their own environment/home situation:

That you can, of course, look behind that person. And that also has added value. Because, in principle, when you come to the practice, we do not get that home situation. So that's really an improvement [ …] People are in their own environment. In their familiar environment. So, you can see that people are also a bit more open and want to talk about it a bit more. (Physiotherapist 3)

In such a way, the forced shift towards remote care was not providing much of an augmented embodied relations with the world, but instead was offering an inferior mediation. Being robbed of the ‘in the flesh’ possibilities of work, physiotherapists shifted to inventing new coping strategies, experimenting with how they could still ensure the performance of care. In what follows, I outline what this coping entailed.

### Coping with the Lockdown

#### Repurposing General Communication Technology

Most physiotherapists recalled that they shifted to providing telecare in a matter of days and that enrolling technology was not perceived by them as problematic. The tools that they used were basic and familiar to them: they used Skype, Zoom, Google Hangouts, and FaceTime, and occasionally used WhatsApp and phone calls to stay in touch with patients. Other physiotherapists signed a contract with a special software provider that offered protection of medically sensitive information but feature-wise only offered basic video calling functionality. Those physiotherapists who shifted to the special software still reported frequently relying on the phone, WhatsApp, and Zoom more often than on the specialised software to consult their patients.

Some physiotherapists reflected on how years ago, telecare in their sector seemed unachievable and was met with resistance. Under crisis conditions, the transition, however, seemed to go smoothly. This was attributed partly to the fact that digital literacy grew over the years and there was a much higher level of adoption of general communication technology. Another reason was attributed to the circumstances that alleviated seemingly persistent barriers to adopting what used to be an inconceivable solution before:

[The transition] actually went very smoothly. The older colleagues also have taken it up very quickly. It also made a difference that the KNGF, the national organization, the Royal Dutch community for physiotherapists, also had a database, which also had some videos with exercises. People could easily find their way around the internet to look it up themselves. And after a few colleagues took it up, it spread like an oil slick. We also have therapists in their 60s, but they also do video calling. The time was right for it, I think. Circumstances forced us to. And then you see that something that has been possible for a long time, but was simply not done, is now just being picked up and just works. It actually got adopted very quickly. (Physiotherapist 3)

Another physiotherapist recalled: ‘We just tried all available solutions that have been around for a long time. Now everyone is digitally literate, so you know how to work with a smartphone, how to work with a laptop. […] So, it all came naturally.’ (Physiotherapist 10)

One could interpret that the quick shift to remote care and characterising it as ‘natural’ and ‘smooth’ is related to the fact that general communication tools, like FaceTime, Skype or WhatsApp have achieved their ‘transparency’ in other areas of everyday life, becoming embedded in the fabric of daily interactions. For example, in recalling their experiences of reductions in embodied relations with patients, physiotherapists did not explicitly refer to any features of technologies: their limitations were not emphasised in the accounts of physiotherapists; they focused on the limitations of their perceptual action instead. Such accounts may be interpreted in postphenomenological terms as illustrating how the tools themselves faded from their awareness and therefore presented themselves naturally and ‘organically’ as a solution to the limitations imposed by the lockdown.

It is also illustrative that the specialised software, although designed directly for the occupational work of physiotherapy and supposedly offered features to benefit medical practices, was quite soon abandoned by the professionals. It quickly became apparent to physiotherapists that because the emphasis of the software was on the features of medical data protection, rather than on supporting the embodied relations with patients, it did not appeal to the concerns of sensory performance that were more important for their work.

#### Recalibrating Senses

In the absence of haptic perception, physiotherapists had to increase their reliance on visual and auditory perception and focus more intensively on interpreting cues available through the online means to sustain embodied relations with patients. As one of them put it: ‘You’ve actually lost a sense. […] If you’re going blind, you’ve got to be better at hearing.’ (Physiotherapist 10). Another one explained:

You don't have your full senses engaged. I just must pay close attention to the screen and the person. What does that person emit? And if you're really thinking about what questions to ask, you're not always looking at the image. Yes, and again, what you normally just pick up in the treatment room, here it is much easier to misunderstand... That is a challenge to turn on and use those antennae in video calling. (Physiotherapist 7)

Without being able to touch or illustrate movements with the whole embodiment, physiotherapists had to increase the verbalization of their otherwise tacit embodied knowledge to the great deal: ‘I think you just have to explain something much more elaborate. Also, in terms of exercises. You can hardly show it, so to speak.’ (Physiotherapist 2).

Another physiotherapist explained how dealing with the lost sense requires a different, lengthier approach of tuning into their patients’ experiences:

The initial screening and anamnesis look different […] And that's difficult because you just didn't feel [patient’s body] yourself. A patient this morning, for example, said “I have a bump in the back of my knee”. And then I ask him “let me see it”. And he then says, “Now you cannot see it, it is only there at the end of the day”. So, because of the answers he gives, you can cross some things out and you'll get at some diagnosis. But you didn't feel it yourself. Sometimes patients really describe it very differently and experience it differently than I would feel it. So that is difficult. (Physiotherapist 9)

Careful verbalization and paying attention to the patient in a new way was mentioned as the key new skill to deal with the loss of haptic perception:

You don't have much of a physical contact, so asking the right questions is very important. You really must ask that patient what his real need for help is. Because for example, if normally you could move patient’s neck with your hands and the patient is in pain, then normally you are feeling yes, that the neck either bounces, or that is very empty, or that is an immediate feeling to your finger, that is locked. So, you get your information from that. So, to obtain such information you have to ask that patient very carefully. (Physiotherapist 5)

The shift from the ‘in the flesh’ relations towards mediated care thus exposed physiotherapists to the richness of the sensory engagement that was characteristic of their work with patients. In traditional practice, they enrolled their senses in a holistic way, relating to their patients directly, tacitly engaging their perceptual skills in the performance of work. The sensory reductions that have been brought forward by the mediation became a trigger for becoming explicitly aware what these perceptual skills entailed and what were the reductions that had to be compensated for to ensure continuity of care.

#### Enrolling Mundane Artifacts and Routines

Another way to cope with reduced embodied experience was to creatively use the available objects or environment in a patient’s home to recreate the haptic or direct engagement with their bodies. For example, one physiotherapist explained that to ensure a more holistic perception of the patient, physiotherapists made do with whatever was available in the vicinity of the patient:

It is very different because I can’t touch the patient. If I needed to check if the shoulder was hurting, I can see if their arm is able to go up, to the side, behind their head. Now I am not able to test how strong they are. So, I instead can ask them to pick up something from the refrigerator. I would ask them to fold some clothes or put on a jacket. And then they will tell me whether some of those parts hurt. So, the evaluation process becomes very limited because I am not able to touch them. (Physiotherapist 4)

The same physiotherapist shared that he enrolled the help of others, such as family members, to manipulate the patients’ environment and obtain otherwise unavailable information: ‘I can have a family member push in the back of the patient somewhere, and with the video exactly showing me where they are hurting, so that I will understand better what’s going on’. Another physiotherapist recalled:

Suppose someone might have torn his anterior cruciate ligament. Yes, that's useful to know. But as a patient, you cannot test it yourself. So, we find ways to just do a little research. What I said to that patient: “Stand up against the wall and push your arm against the wall.” And that's actually a test of strength for us. (Physiotherapist 1)

One physiotherapist also discovered:

The first two sessions, you have to look at the patient’s home and see where the best place for them is to exercise. So, the first two times it’s a learning process. If they have any weights, I tell them, “Next time make sure you have them next to you, so we can start right away”. (Physiotherapist 4)

These experiences illustrate how tools and artifacts exhibit their multistability when skilled sentient practitioners put them to creative uses in novel combinations. As Ihde (2009) reflects in his example of archery, the ‘same practice’ of archery can be performed radically differently and exhibit different materialities depending on the skilled bodily performances in situated contexts. Similarly, the physiotherapy practice transformed in the lockdown by enrolling different materialities: rather than specialised equipment, it turned to rely on mundane objects, such as fridges, walls, and foldable laundry. Those objects, along with the Zoom or Skype software features, are not strictly speaking tools for performing medical care but can emerge as such during experimentation combining the physiotherapeutic bodily skills, attuned to perceiving the patient symptoms and the improvisational ways of finding alternative methods of sensing and tuning into the patient’s body in the absence of direct perception.

#### Embodied Knowledge Externalised and Transferred to Patients

A variety of ways in which physiotherapists engaged technology for providing remote care also included other tools which they could employ to support care. In addition to general communication technologies, physiotherapists enrolled cameras on their phones for filming short videos, YouTube video clips and professional databases, all to support their treatment at a distance. One shift that followed such experimentation with remote care was the emergence and increase of codified embodied knowledge. Many physiotherapists engaged in creating a database of videos to support the video consults, which then became used as a support of customary practice: ‘From day one, our therapists actually started to create videos and exercises. And we made kind of our own database out of that.’ (Physiotherapist 3). The exercise schemes were created and sent by the physiotherapists among all organizations. Another physiotherapist said:

If I have to do an exercise or something, then I have to film it from different angles or send videos. For example, I have a technique that I want to teach someone that I would normally do before. Then I sent them YouTube videos. And then he looked at it again. And with me on camera. That way, you get creative. So that was a nice replacement for what I would otherwise experience here in the treatment room. (Physiotherapist 2)

Externalising and codifying embodied knowledge also meant involving the patient much more centrally in the care process, because there was no direct haptic manipulation possible, which unintendedly meant making the patient responsible for their own body. A physiotherapist reflected on the novel skills it required for engaging in interaction:

It is important to formulate concrete questions that a patient also understands. I explain a lot with my hands, also on a video call. I can grab a knee joint, so I can visually demonstrate things to the patient. Like, "That's the way it is, and can you now point out how it is?" You should also have some empathy to the patient, to translate it into laymen language, so that the patient also understands. For example, in a meniscus test, someone must stand on one leg and then they have to turn. The foot must then remain standing so that they understand it well and execute it properly. You should try to include those people as best you can”. (Physiotherapist 9)

Another one referred to a similarly embodied knowledge externalisation:

So you have to do that more consciously, and you also have to look a lot more to see if it is well understood by the patients. So basically, that's kind of a teaching because it's something the patients now must do themselves anyway. For example, with the following week, if you then ask, “Would you tell me what I explained to you last week?” then that is usually not well explained to you. So that's always interesting if that's well understood. (Physiotherapist 8)

Externalising knowledge and transferring it to patients implied performing yet another embodied practice of relating to patients. For one, it may be seen as further ‘disembodied’ practice: Recording videos and exercises are done for potentially wider sets of audiences and can be taken out of their intimate ‘in the flesh’ relation context and be potentially re-used and re-played by many anonymous others. The laborious transfer of own haptic skills to patients can also imply relating to patients as different recipients of care: The ones who now had to be conceived as active sensory beings themselves, activating their own perceptual skills for helping themselves in the absence of the skilled practitioner to take on their customary role.

### Emerging Consequences

#### Extending Rather than Replacing Care

A variety of ways in which physiotherapists engaged technology for providing remote care triggered nuanced reflections about when and which types of services could be enhanced by online tools and which ones are less amenable to it. Many reflected that one could think of customised scenarios and solutions of when to use the mediated interaction and in which combination, as a complement to in the flesh presence. In the words of one of them:

We can do some of the services online. Also, in the first line. You can do exercises. For example, we have a paediatric physiotherapist, and he does that very nicely with mother and child on the other side of the video. And there is also a nice collaboration where parents and children make a film and then send it back. So, there you can see where possible, care is being digitized and relies on video calling. (Physiotherapist 3)

Another one reflected on the specific cases where online tools can help increase access to particular groups:

And that is something that physiotherapy will now take into account, to what extent can we use the video calls to people who are more vulnerable or difficult to reach, to see them and then actually be able to help remotely. And who knows for what else! For example, some people don't need contact treatment. You can often then offer a person: ‘We are going to treat this with exercises’. Or, for example, for people who are in a certain specialized hospital to treat them remotely. So, I definitely see possibilities for that, but we never really used it for remote care. (Physiotherapist 6)

Extending care was also envisioned to be done via performing initial consultations online and then following up with ‘in the flesh’ presence. One physiotherapist discovered how such a combination of embodied relations worked for him:

For example, I had a patient with shoulder complaints, and he had already filled in some things via intake form. I then first reviewed it. I then called him, and it soon became apparent that a certain type of complaint was actually in the shoulder. So, I gave some instructions and some practice assignments. Then the following week I agreed to make a video call to watch. Called again the following week. Then it turned out that things were not going well and that the complaints had increased again. So, all this gave me doubts about the first diagnosis I gave at the time. So, then we quickly decided to schedule him to be physically see what's going on. That's a bit of the extra triage step. I see the added value in that. As a potential keeper. (Physiotherapist 8)

These new enthusiastic ideas about a variety of ways in which online care could potentially be performed in the future illustrate the consequences of direct experience of physiotherapists engaging in remote work. Whereas telecare used to be seen as an indisputably unwelcome reduction (Reinhardt et al., 2021) it could now also be imagined as an alternative stability of physiotherapeutic practice, existing side-by-side with another dominantly stable performance. The programs currently running for stimulating e-consultations in medicine are hoping to benefit from the knowledge obtained from this direct perceptual engagement as they have acquired more firsthand experience of what the possibilities are (e.g., Fahy and Williams, 2021).

#### Exhausted Bodies on Zoom

Perhaps counterintuitively, almost all physiotherapists shared that the shift towards remote care was experienced by them as bodily exhausting and draining energy:

So to benefit and to really have an advantage of the treatment via video calling, there is simply extra effort involved. And I also hear from many people, and I think that myself, that it is more labor intensive. Because you're missing some things anyway. And yes, you should pay much more attention to the words now. While normally as a therapist, you pay much more attention to someone's movement and his body. (Physiotherapist 3)

Another one shared: ‘It also takes a lot more energy and effort than just seeing them in practice.’ (Physiotherapist 2). In the words of another one:

When I move and I feel the patient’s body, I get so much feedback from that. So much literal interaction. [Remote care] costs me many times more energy. I am normally never tired when I come home from work. Really never tired. Even though I have a physical profession. I miss that. Now I don't sleep well at night because of [not being able to do it]. (Physiotherapist 6)

The theme of bodily exhaustion from having to interact online with limited sensing opportunities was very pronounced and emotional for all physiotherapists, speaking of their desire to get back to the ‘in the flesh’ experience of care. In postphenomenological terms, such reflections can be interpreted in a sense of little attractiveness that is offered by the mediation that does not present itself as desirable for augmenting senses at work. In contrast, for example, to other tools, such as robotic apparatus for surgery (Sergeeva et al., 2020), mediated care via video calling did not appeal as something magnifying embodied experience in an attractive or unprecedented way.

Another note on the bodily exhaustion theme is perhaps a reminder of the fact that there is a physical body involved in using such tools as Zoom or FaceTime, which sometimes disappears in the general understanding of performance of the online or remote work. Trying to communicate embodied knowledge through the screen is an embodied activity too, and the exhaustion from sitting and interacting with limited sensing opportunities could serve as an important warning for building tools and work configurations that are sensitive to the embodiment and in the flesh experience of users, paying attention of how the online solutions figure in mediating the embodied relations with the world.

## Discussion and Conclusion

The transformations described in the vignette are not unique to physiotherapy settings but seem to emerge in other settings as well. Education is one example, where similar concerns have surfaced around online course delivery: The direct embodied engagement of teachers and students replaced by mediated relations has been both exciting and depleting for many with current efforts to devise novel hybrid formats that extend rather than replace the ‘on campus’ teaching (Gerhardt and Russo, 2021; Razavi, 2020). Reflections on academic conferences provide another illustration. Online gatherings have resulted in some augmentations: Facilitating inclusion and opportunities to speak up for previously marginalised participants, alleviating the need for travel, offering shorter and diverse formats of presentations while simultaneously giving rise to Zoom fatigue, body aching and longing to return to the ‘exhilaration’ experienced from ‘serendipitous encounters’, and otherwise ‘hedonic’ experiences of live conferencing (Etzion et al., 2021). The postphenomenological perspective offers a way to make sense of the emotional ambiguity of such transformations: seeing it as simultaneously empowering and exhausting, exciting and dreadful, futuristically attractive, entrepreneurial, and yet longing for the simpler times when (supposedly) no mediation put a limit to our direct engagements with each other.

With the lockdown analysis as an illustration, this essay also hopes to inspire future CSCW researchers to take on postphenomenological concepts in their studies of work. Undoubtedly, within the CSCW field, the focus on lived experience and situated conduct ‘in the wild’ is already a widely accepted ontological commitment, e.g., serving as a backdrop for detailed workplace studies and ethnomethodological studies of work (Luff et al., 2000; Rouncefield and Tolmie, 2011). The focus on embodied action and interaction is an important sensitivity for those analytical orientations (e.g., De Carvalho et al., 2018; Heath and Luff, 1992; Hindmarsh and Heath, 2000a; Hindmarsh and Pilnick, 2007). Postphenomenology can bring a complementary insight to those studies in that it takes a somewhat different stance towards embodied action than typically examined in CSCW. Its focus on the *transformation* of human perception and insistence on identifying the augmentations and reductions that always accompany technological mediation is an analytical focus than is different to the one, e.g., typically considered in ethnomethodology.^4^ The augmentation-reduction analysis can bring into focus some previously unreflected upon aspects of sensory and perceptual experiences that are lost with the mediation but often do not surface for immediate examination if not explicitly interrogated.

Postphenomenology’s concern with the *affective* stance—longing for the promises of augmentation and rejecting or forgetting the reductions—may be another conceptual sensitivity inspiring future studies. While we have a rich array of workplace studies on bodily conduct in the performance of practice, e.g., including the role of gesturing, gazing, orienting, posture, and shared use of artifacts (Fischer et al., 2013; Heath and Luff, 1992; Hindmarsh and Heath, 2000a), we have fewer insights into how the augmentations and reductions provided by novel tools are experienced emotionally by users in situated practices. The sentiments of physiotherapists who were both excited to be able to continue their work online and yet dreaded the depleting character of such a practice are an illustration of such contradictory emotions associated with the technological mediation of perception.

In line with the key objective of CSCW of ‘understanding the nature and requirements of cooperative work with the objective of designing computer-based technologies for cooperative work arrangements (Schmidt and Bannon 1992, p. 11), the postphenomenological perspective offered here may also inform guidelines for technology development. The design of new technological mediations, especially those that involve technologies changing embodied relations at work can focus on evaluating how it performs and participates in mediating human-world relationships. For example, virtual reality, immersive presence, drones, or robotics are examples of technology mediating direct engagement and presence at the site of work and ability to manipulate the environment at a distance, affecting senses of human touch, dexterity, range of vision and auditory perception. Those technologies are at the forefront of what is envisioned for the future of work, as also illustrated in attempts to utilize them to deal with the crisis (Singh et al., 2020; Somauroo, 2020). Given that previous research has often been more occupied with technologies supporting information transfer, and less on technologies directed at embodied action, we have relatively poor conceptual vocabulary to analyse the transformation made possible by such tools; and the perspective in this essay offers one way forward.

One specific way in which design could borrow from postphenomenology is by attending to how technology helps attain ‘transparency’ and what kinds of ‘multistabilities’ it offers. One way to import those concepts could be, for example, by envisioning transparency or multistability as evaluation criteria in design: striving for transparency or appeal of technological augmentations to such an extent that reductions fade from focal awareness. The physiotherapy vignette provided here offered an example of the primarily reduction of sensory experience; however, there are cases where the augmentation of performance has been much more appealing to users. For example, in a robotic surgery case, the augmentation that was offered by the Da Vinci Surgical System robot—magnified vision, alleviating bodily exhaustion, offering superior dexterity—far outweighed the reduction of haptic perception or peripheral awareness and, therefore, explained why users were eager to embrace technology despite significant reductions it offered (Sergeeva et al., 2020). The analysis and design of other supportive tools can thus focus on juxtaposing the augmentations and reductions that are offered by the tool and evaluating whether the augmentation is likely to be more appealing for the work purposes than the reductions.

Postphenomenology may also provide a complementary perspective on such central concerns in the CSCW field as matters of achieving shared or mutual awareness, overcoming distance, and establishing sense of presence. A rich body of studies, starting with the traditional time—space matrix of Johansen (1988), has engaged with exploring the question of how best support distributed work, and how tools can help achieve the sense of ‘being there’ or even ‘beyond being there’ (Hollan and Stornetta, 1992), which in postphenomenological terms, would be regarded as an augmentation of embodied relations. Some early studies of distributed work have problematized the ‘death of distance’ thesis, arguing for the persistent importance of co-location and assessing the impact of co-location on productivity (Hinds and Kiesler, 2002; Olson and Olson, 2000). Those studies sometimes concluded that co-location remained as important as ever and virtual solutions could rarely live up to the proximity alternative while other voices problematized the aim of replicating the physical world in the virtual space (Fitzpatrick et al., 1996). A postphenomenological perspective can join these conversations by reconsidering ‘distance’ from the perspective of embodied relations and sensory engagement: When the embodied relations become the unit of analysis, the distance reappears not as a spatial dimension but rather as a perceptual dimension: how experiential engagement in terms of feeling, hearing, manipulating, seeing is configured with the tools.

Work transformations that have occurred during the pandemic, illustrated by this vignette through the postphenomenological reading, can be seen to provide a window into the lessons learned as a result of forced experimentation with practices. The ways in which physiotherapists recalibrated their sensory engagement at work, when using one essential sense was no longer possible, exposed their reliance on the sensory apparatus and brought into relief how much embodied knowledge was at work, which was something they were not explicitly aware of before the crisis. The coping strategies also revealed novel dimensions of their skill and practices—imagining new scenarios for remote care, compensating for the lost senses via externalisation, codifying tacit embodied knowledge. These experimentations also revealed to them some scenarios where the haptic senses may be not essential and specializations that can be usefully complemented by technological mediation. Such lessons learned through first hand experience may teach us that arranging a space for some experimentation may be one way to develop user-centred design scenarios.

In sum, this essay introduced postphenomenology as a perspective to join the extensive body of studies in CSCW committed to investigating the production of intelligible conduct in situ (Crabtree et al., 2012; Luff et al., 2000; Suchman, 1987) by zooming into the bodily and perceptual apparatus as the unit of analysis. Its careful attention to the non-neutrality of how technologies mediate experience can usefully reveal the emotional significance of technological mediations for actors involved, since every amplification and reduction comes with its own emotional appeal. Finally, interrogating the multiple stabilities of everyday technologies and their alternative uses may offer a way of exploring alternative experimentations and reimagining perhaps mundane tools in novel contexts.

## Footnotes

1 For example, comparing modern technology with the ‘older handwork technology,’ which he considers ‘completely different and therefore new’ (p. 5), Heidegger writes: ‘One can object that it [unconcealment] indeed holds for Greek thought and that at best it might apply to the techniques of the handcraftsman, but that it simply does not fit modern machine-powered technology. And it is precisely the latter and it alone that is the *disturbing* thing, that moves us to ask the question concerning technology per se. It is said that modern technology is something incomparably different from all earlier technologies because it is based on modern physics as an exact science. […] The revealing that rules in modern technology is a challenging *[Herausfordern]*, which puts to nature the unreasonable demand that it supplies energy that can be extracted and stored as such’ (p.14, emphasis added).

2 For a comprehensive analysis and critique of Heidegger’s take on technology, see e.g., Chapter 2 in Verbeek (2005) ‘What Things Do’.

3 Data was collected with the help of a research assistant who was also involved in the first rounds of analysing the interviews

4 For example, Paul Dourish’s approach is characteristic of such differences in thinking about embodied action. Dourish is explicitly distinguishing his approach to embodiment from (among others) the postphenomenological interest in embodied relations: ‘I use the term [embodiment] to capture a sense of “phenomenological presence”, the way that a variety of interactive phenomena arise from a direct and engaged participation in the world. […] However, in Merleau-Ponty’s work, the idea of embodiment is used to draw particular attention to the *role of the body*. This concern with the body is echoed in much current work in Critical Theory, and particularly in exploration into the relationship between questions of self and technology, such as the “cyborg” work initiated by Donna Haraway (1991), Stone’s (1991) comments on virtual presence, or (more distantly) Don Ihde’s (1991) investigations of the mediating role of technology in science. Although I am sympathetic to their perspectives, however, my concerns here are not those of Haraway and her colleagues, *nor should my use of the term embodied be confused with the issues that they wish to identify’* (2001, p. 115, emphasis added)
